# Comparison of fasting and random lipid profiles among subjects with type 2 diabetes mellitus: an outpatient-based cross-sectional study in Bangladesh

**DOI:** 10.1186/s13098-023-01120-y

**Published:** 2023-06-26

**Authors:** A. B. M. Kamrul-Hasan, Samir Kumar Talukder, Md Ahamedul Kabir, Marufa Mustari, Md Masud Un Nabi, Abu Jar Gaffar, Md Firoj Hossain, Muhammad Shah Alam, Md Rashedul Islam, Mohammad Abdul Hannan, Nusrat Zarin, Ajit Kumar Paul, Farhana Akter, Afsar Ahammed, Md Lutful Kabir, Mohammad Motiur Rahman, Md Asaduzzaman, Mohammad Saifuddin, Palash Kumar Chanda, Md Abdur Rafi, Mohammad Jahid Hasan, Shahjada Selim

**Affiliations:** 1 Department of Endocrinology, Mymensingh Medical College, Mymensingh, Bangladesh; 2 Department of Endocrinology, Rangpur Medical College, Rangpur, Bangladesh; 3 Department of Endocrinology, TMSS Medical College, Bogura, Bangladesh; 4 Department of Endocrinology, Bangabandhu Sheikh Mujib Medical University, Dhaka, Bangladesh; 5 Department of Endocrinology, Rajshahi Medical College, Rajshahi, Bangladesh; 6 Department of Pathology, Naogaon Medical College, Naogaon, Bangladesh; 7 Department of Endocrinology, Mugda Medical College, Dhaka, Bangladesh; 8 Department of Medicine, Army Medical College Cumilla, Cumilla, Bangladesh; 9 Department of Neurology, BIRDEM General Hospital, Dhaka, Bangladesh; 10 Department of Endocrinology, North East Medical College, Sylhet, Bangladesh; 11 Department of Endocrinology, Bangladesh Institute of Health Sciences, Dhaka, Bangladesh; 12 Department of Endocrinology, Mainamoti Medical College, Cumilla, Bangladesh; 13 Department of Endocrinology, Chittagong Medical College, Chittagong, Bangladesh; 14 Department of Endocrinology, National Institute of Traumatology and Orthopaedic Rehabilitation (NITOR), Dhaka, Bangladesh; 15 Department of Endocrinology, Rangpur Medical College, Rangpur, Bangladesh; 16 Department of Medicine, Rajshahi Medical College Hospital, Rajshahi, Bangladesh; 17 Department of Endocrinology, Shaheed Sheikh Abu Naser Specialized Hospital, Khulna, Bangladesh; 18 Department of Endocrinology, Dhaka Medical College, Dhaka, Bangladesh; 19 Department of Endocrinology, Mymensingh Medical College Hospital, Mymensingh, Bangladesh; 20 Pi Research Development Center, Dhaka, Bangladesh

**Keywords:** Fasting lipid profile, Random lipid profile, T2DM, Cardiovascular risk, Lipid-lowering therapy

## Abstract

**Background:**

Despite the wide acceptability of fasting lipid profiles in practice, emerging evidence suggests that random lipid profiles might be a convenient alternative for lipid measurement. The objective of the present study was to compare the fasting and random lipid profile among subjects with type 2 diabetes mellitus (T2DM).

**Methods:**

The present cross-sectional study included 1543 subjects with T2DM visiting several endocrinology outpatient clinics throughout Bangladesh from January to December 2021. The fasting lipid profile was measured in the morning following 8–10 h of overnight fasting, and the random lipid profile was measured at any time of the day, irrespective of the last meal. The values of fasting and random lipids were compared using the Wilcoxon signed-rank test and Spearman rank correlation coefficients.

**Results:**

In this study, a good level of correlation was observed between fasting and random lipid levels [r = 0.793, p < 0.001 for triglyceride (TG); r = 0.873, p < 0.001 for low-density lipoprotein cholesterol (LDL-C); r = 0.609, p < 0.001 for high-density lipoprotein cholesterol (HDL-C); and r = 0.780, p < 0.001 for total cholesterol (TC)]. In addition, TG and TC levels increased by 14% and 0.51%, respectively, in the random state compared to the fasting state (p- <0.05), while LDL-C levels decreased by 0.71% (p-value 0.42). No change was noticed in the HDL-C level. The difference between fasting and random lipid profiles was similar irrespective of patients’ age, sex, BMI, glucose-lowering drug(s), and lipid-lowering therapy.

**Conclusions:**

Random lipid profile correlates significantly with fasting lipid profile with little difference. Hence, it might be a reliable alternative for fasting lipid profile in patients with T2DM.

## Background

In current clinical practice, the fasting lipid profile is commonly used to diagnose and manage dyslipidemia [1]. However, this procedure is often challenging for specific groups of patients, including those suffering from type 2 diabetes mellitus (T2DM) and on insulin or intensive oral hypoglycemic therapy due to the increased risk of hypoglycemia after prolonged fasting. Moreover, it is not feasible to convince patients for multiple visits for lipid testing. This limits the diagnosis and therapeutic intervention for dyslipidemia in T2DM patients and consequently increases cardiovascular risk in the long run [2].

Considering these limitations of the fasting lipid profile test, evaluation of random lipid profiles is suggested by several recent guidelines of professional bodies and societies as an alternative for diagnosing and monitoring dyslipidemia. These guidelines are grounded on the evidence that random lipid profiles strongly correlate with fasting lipid profiles in the general population and T2DM patients, irrespective of statin therapy [3–5]. Moreover, a random lipid profile can also predict the risk of cardiovascular morbidities and fasting lipid profiles in these patients [6–8]. Based on this emerging evidence of the potentiality of random lipid profiles as a suitable alternative to the fasting lipid profile, the Danish Society for Clinical Biochemistry recommended this test as routine practice for their national laboratories in 2009. Since then, several other societies have accepted it as a routine screening test [9]. For example, the European guidelines recommended that fasting lipids are not routinely required to assess or monitor lipid-lowering therapy [10]. The American guidelines recommended that the random lipid profile might be an acceptable alternative for baseline lipid assessment in patients not yet on statin therapy [11].

Despite these recommendations, random lipid profile measurements still need to be universally applicable, and additional fasting lipid testing is suggested in certain clinical conditions. For example, the European consensus has recommended a further reassessment of fasting triglyceride (TG) levels if random TG levels are ≥ 350 mg/dl as TG concentration is more stable in the fasting state [10]. Another concern regarding random lipid profile measurements is that these recommendations are not exclusive to T2DM patients. Insulin resistance in T2DM patients can significantly raise triglycerides (TGs) and lower high-density lipoprotein cholesterol (HDL-C) levels and ultimately increase atherosclerotic risks [12].

A few studies were conducted to assess the credibility of random lipid profiles as an alternative to fasting lipid profiles in patients with T2DM. A study from Singapore reported that a random lipid profile was compatible with the fasting lipid profile in Asian T2DM patients on lipid-lowering agents [13]. However, the findings of this study might not be inferential for the overall T2DM patient population, as it did not include patients who were not currently on lipid-lowering therapy. Hence, we conducted the present study to compare the fasting and random lipid profile among Bangladeshi subjects with T2DM, irrespective of their glucose-lowering or lipid-lowering treatment.

## Methods

### Study setting and participants

A cross-sectional study was conducted at several endocrinology outpatient clinics throughout Bangladesh between January and December 2021. Diabetes clinics of fifteen tertiary care teaching hospitals from eight administrative divisions of the country were included in the present study, namely, Mymensingh Medical College Hospital, Bangabandhu Sheikh Mujib Medical University, Dhaka Medical College Hospital, Bangladesh Institute of Health Sciences, BIRDEM General Hospital, Mugda Medical College Hospital, Rangpur Medical College Hospital, TMSS Medical College Hospital, Chattogram Medical College Hospital, Cumilla Diabetic Hospital, Rajshahi Medical College Hospital, Naogaon Medical College Hospital, North East Medical College Hospital, Shaheed Sheikh Abu Naser Specialized Hospital, and Sher-e-Bangla Medical College Hospital.

The sample size for the present study was calculated from the following formula: n = z^2^p(1-p)/d^2^, where p = estimated proportion (considered as 0.5 for the present study), d = precision of error (considered as 0.3 for the present study). The formula provided a sample size of 1067 for the study. Considering a design effect of 1.5, the final sample size was 1600.

Convenient sampling was used to recruit the participants according to the inclusion and exclusion criteria. The inclusion criteria for the present study were adult patients (aged ≥ 18 years) of either gender, newly or previously diagnosed with T2DM, irrespective of lipid-lowering therapy. Exclusion criteria were (i) patients with type 1 diabetes mellitus, (ii) pregnant women, (iii) patients who required emergency hospital admission due to diabetes-related or other causes, and (iv) patients who were terminally ill. In this study, T2DM was defined by American Diabetes Association (ADA) criteria: HbA1c ≥ 6.5% or fasting plasma glucose (FPG) ≥ 126 mg/dL (7 mmol/L) or 2-hour plasma glucose ≥ 200 mg/dL (11.1 mmol/L) during an oral glucose tolerance test (OGTT) or in a patient with classic symptoms of hyperglycemia or hyperglycemic crisis, a random plasma glucose ≥ 200 mg/dL (11.1 mmol/L) [14]. Good glycemic control was defined as HbA1c < 7% [14].

### Data collection procedure

Patients’ sociodemographic, clinical, and laboratory-related information was collected by investigators using a structured questionnaire. This information included patients’ age, sex, area of residence, body mass index (BMI), duration of diabetes, anti-diabetic drugs, glycemic control, self-reported comorbidities like hypertension, thyroid disorders, respiratory diseases, etc. complications of diabetes, use of a lipid-lowering agent, etc. Sociodemographic and disease-related information were collected through face-to-face interviews. Hypertension was defined as systolic blood pressure ≥ 140 mmHg or diastolic blood pressure ≥ 90 mmHg and/or currently taking antihypertensive drugs and/or self-reported history of hypertension and taking antihypertensive drugs. Body mass index (BMI) was calculated by height and weight and categorized as underweight (< 18.5 kg/m^2^), normal (18.5–22.9 kg/m^2^), overweight (23-27.4 kg/m^2^), and obese (≥ 27.5 kg/m^2^) according to the WHO cutoff values for the Asian population [15].

### Laboratory measures

Patients received their clinical follow-ups on the day of the first visit and were appointed for a fasting lipid profile on the next day of clinical consultation. Fasting blood samples were collected on the appointed day following overnight (8 to 10 h) fasting [16]. Random blood samples were collected during physician visits at any time of the same day, irrespective of the last meals taken by the study subjects. From the fasting and random blood samples, lipid profiles, including triglyceride (TG), low-density lipoprotein cholesterol (LDL-C), high-density lipoprotein cholesterol (HDL-C), and total cholesterol (TC) were analyzed using an automatic analyzer.

### Statistical analysis

All statistical analyses were performed using STATA version 17.0. Continuous variables were presented as the mean with standard deviation (SD) for normal distribution or median with interquartile range (IQR) for non-normal distribution. Categorical variables are expressed as numbers (percentages). Spearman rank correlation coefficients (r) were used to assess correlations between fasting and random lipid profiles. Fasting and random lipid profiles were compared using a nonparametric test of two paired samples (Wilcoxon signed-rank test). The difference (%) of lipid panels was calculated as follows: Diff (%) = (random − fasting)/fasting ×100, and it was compared among different groups of patients by the Mann–Whitney U test. Bland–Altman analysis was conducted to assess agreement between fasting and random lipid tests. All tests were two-sided and defined statistical significance by p-value < 0.05.

## Result

### Baseline characteristics

A total of 1543 T2DM patients were enrolled in the present study. Their mean (SD) age was 46 (12.5) years, and approximately 57% were female. The duration of T2DM in most patients was between 0 and 10 years. Almost one-third were on insulin therapy, while others were on oral hypoglycemic agents (OHA). However, nearly one-third of the patients (33%) had good glycemic control. More than half of the patients (59%) had comorbid hypertension. Among the microvascular complications, diabetic neuropathy was the most prevalent among almost 46% of patients, while macrovascular complications such as cardiovascular disease were present in 37%. Nearly 59% of the patients were on lipid-lowering therapy (Table 1).

**Table 1:** Baseline characteristics of the T2DM patients (n = 1543)

Characteristics	n	%
Age (years) (mean = 45.91, SD = 12.48)		
< 45	805	52.17
≥ 45	738	47.83
Sex		
Male	664	43.03
Female	879	56.97
Residence		
Rural	730	47.31
Urban	813	52.69
Duration of T2DM (years)		
0 to 10	1204	78.03
> 10	339	21.97
Insulin Intake		
Yes	571	37.01
No	972	62.99
Comorbidity		
Hypertension	908	58.85
Hypothyroidism	156	10.11
Hyperthyroidism	35	2.27
Bronchial asthma	174	11.28
Complications of T2DM		
Neuropathy	709	45.95
Nephropathy	331	21.45
Retinopathy	222	14.39
Ischemic heart disease	573	37.14
Peripheral vascular diseases	300	19.44
Stroke	198	12.83
Lipid lowering agent		
No	628	40.70
Yes	915	59.30
Body mass index (BMI) (kg/m^2^)		
Normal (18.5–23)	187	12.12
Overweight (23-27.5)	769	49.84
Obese (≥ 27.5)	587	38.04
Glycemic control		
Yes (HbA1c < 7)	504	32.66
No (HbA1c ≥ 7)	1039	67.34

* T2DM: Type 2 diabetes mellitus, BMI: Body mass index

### Changes and correlations between fasting and random lipid profile

A significant difference was observed between fasting and random levels for TG and TC (p-value < 0.05). An absolute increase of 26 mg/dl (14%) was observed in the random level of TG compared to the fasting state, and an absolute increase of 1 mg/dl (0.51%) was observed in the random level of TC compared to the fasting state. On the other hand, the level of LDL-C was decreased by 0.71% in the random state compared to the fasting state, although it was not significant (p-value 0.42). The level of HDL-C did not change between these states **(**Table 2**).**

**Table 2:** Comparison between fasting and random lipid profile among subjects with T2DM

Lipids	Fasting	Random	Absolute difference	Diff (%)	p-value for Diff (%)*
	Median (Q1 –Q3)	Median (Q1 –Q3)	Median (Q1 –Q3)		
TG	185 (150 to 245)	226 (178 to 288)	26 (9 to 64)	13.81%	< 0.001
LDL-C	87 (59 to 120)	86 (58 to 120)	-1 (-9 to 7)	-0.71%	0.419
HDL-C	42 (36 to 48)	42 (36 to 48)	0 (-4 to 3)	0.0%	0.208
TC	195 (170 to 230)	200 (170 to 230)	1 (-10 to 13)	0.51%	0.031

*Wilcoxon signed-rank test; TG: triglyceride, LDL-C: low-density lipoprotein cholesterol, HDL-C: high-density lipoprotein cholesterol, TC: total cholesterol

**Fig. 1:**
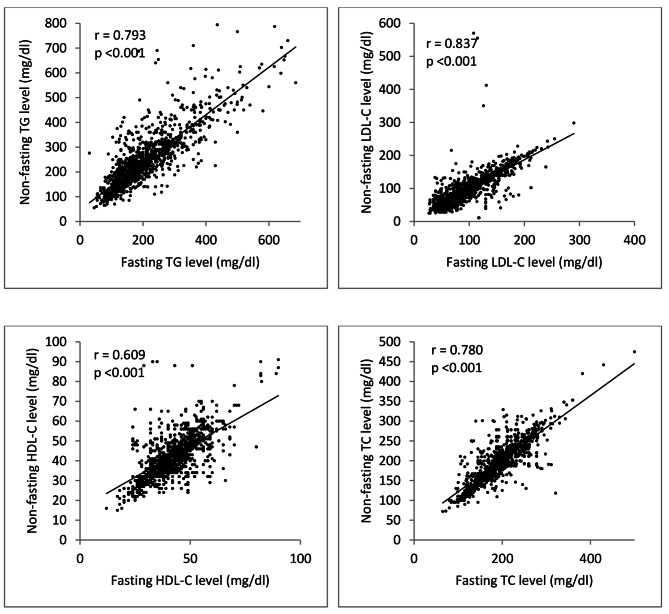
Correlations between fasting and random lipid profile of T2DM patients (r = Spearman correlation coefficient, TG: triglyceride, LDL-C: low-density lipoprotein cholesterol, HDL-C: high-density lipoprotein cholesterol, TC: total cholesterol)

Spearman correlation analysis showed that fasting lipid levels were well correlated with random lipid levels (r = 0.793, p < 0.001, r = 0.873, p < 0.001, r = 0.609, p < 0.001 and r = 0.780, p < 0.001 for TG, LDL-C, HDL-C, and TC respectively) **(**Fig. 1**).**

**Fig. 2:**
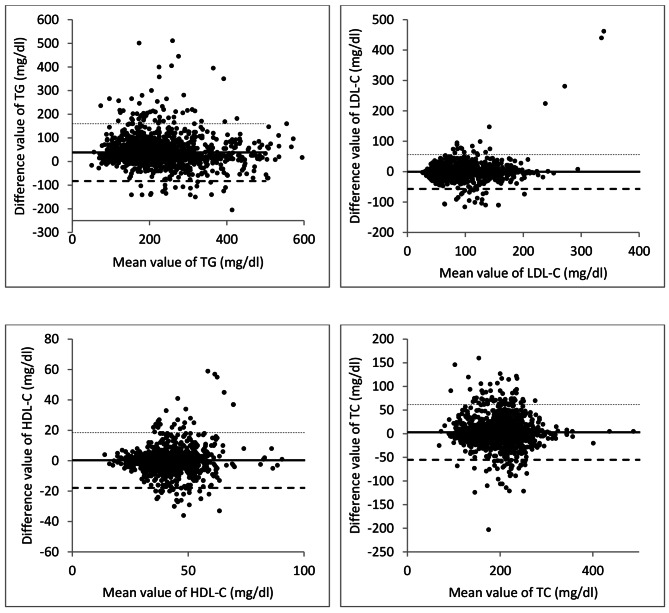
The Bland-Altman plot of lipid parameters of T2DM patients. The plot displays the mean difference and limits of agreement (LoA). Confidential intervals for LoA are shown as hidden line. (TG: triglyceride, LDL-C: low-density lipoprotein cholesterol, HDL-C: high-density lipoprotein cholesterol, TC: total cholesterol)

The Bland–Altman analysis explained that the difference between fasting and random levels of lipids was within the total acceptable error allowed for each lipid parameter **(**Fig. 2**).**

The differences between fasting and random lipid profile (all of the TG, LDL-C, HDL-C, and TC) were similar in different groups of sociodemographic variables (age and sex), BMI, glycemic control and therapeutic modality (anti-diabetic drugs and lipid-lowering agents) **(**Table 3**).**

**Table 3:** Comparison between fasting and random lipid profile of the participants and its relation with patient characteristics

Characteristics	Fasting	Random	Absolute difference	Diff (%)	p-value for Diff (%)*
	Median (Q1 –Q3)	Median (Q1 –Q3)	Median (Q1 –Q3)		
TG					
Sex					
Male	188.5 (150 to 243)	230 (180 to 286)	25 (8.5 to 62)	13.72%	0.498
Female	180 (149 to 246)	223 (178 to 289)	26 (9 to 65)	13.81%	
Age					
< 45	180 (155 to 241)	223 (178 to 282)	28 (8 to 62)	15.06%	0.507
≥ 45	190 (145 to 249)	230 (180 to 289)	25 (9 to 65)	12.5%	
DM drug					
Insulin	195 (150 to 252)	230 (180 to 290)	25 (7 to 66)	12.65%	0.482
OHA	180 (149 to 241)	223 (178 to 286)	26 (9 to 62)	14.21%	
BMI					
Normal	180 (143 to 250)	217 (167 to 288)	20 (6 to 54)	11.43%	0.364
Overweight	180 (150 to 248)	230 (180 to 291)	27 (9 to 66)	14.21%	
Obese	190 (155 to 241)	224 (180 to 280)	26 (9 to 62)	14.28%	
Lipid lowering agent					
No	191 (145 to 266)	230 (180 to 313)	25 (9.4 to 64.5)	12.68%	0.500
Yes	180 (155 to 240)	225 (178 to 270)	26 (8 to 64)	14.28%	
LDL-C					
Sex					
Male	91 (65.5 to 120)	89 (62 to 119)	-1 (-12.38 to 6)	-1.03%	0.457
Female	83 (53 to 120)	83 (56 to 121)	0 (-8 to 8)	0.00%	
Age					
< 45	79 (53 to 113)	79 (51 to 114)	-2 (-10 to 8)	-0.19%	0.502
≥ 45	95 (66 to 129)	95 (65 to 126)	-1 (-9 to 6)	-0.95%	
DM drug					
Insulin	79 (60 to 115)	79 (59 to 114)	-1 (-10 to 10)	0.61%	0.512
OHA	92 (59 to 121)	91 (57 to 123)	-1 (-9 to 6)	-0.87%	
BMI					
Normal	102 (79 to 133)	107 (83 to 130)	1 (-5 to 8)	1.33%	0.472
Overweight	77 (53 to 114)	76 (52 to 113)	-2 (-11 to 7)	-1.75%	
Obese	93 (63 to 123)	90 (59 to 126)	0 (-10 to 6)	0.00%	
Lipid lowering agent					
No	110 (89 to 139)	112 (89 to 139)	0 (-7 to 6)	0.00%	0.520
Yes	68 (49 to 98)	67 (49 to 95)	-1 (-11 to 9)	-1.05%	
HDL-C					
Sex					
Male	41 (35 to 46)	40 (34 to 46.85)	0 (-3 to 3)	0.00%	0.458
Female	43 (37 to 49)	43 (37 to 50)	0 (-4 to 4)	0.00%	
Age					
< 45	43 (36.6 to 49)	43 (36 to 50)	0 (-4 to 5)	0.00%	0.526
≥ 45	41 (36 to 48)	(35 to 48)	-1 (-3 to 3)	-2.02%	
DM drug					
Insulin	42.8 (36 to 48)	43 (36 to 49)	0 (-4 to 4)	0.00%	0.532
OHA	42 (36 to 48)	41 (35 to 48)	-1 (-4 to 3)	-1.98%	
BMI					
Normal	41 (36 to 47)	40 (34 to 47)	-1 (-4.2 to 3)	-3.33%	0.522
Overweight	43 (36 to 49)	43 (36 to 49)	0 (-4 to 4)	0.00%	
Obese	42 (36 to 48)	41 (36 to 48)	0 (-3 to 3)	0.00%	
Lipid lowering agent					
No	40 (35 to 47)	40 (35 to 45)	-1 (-3 to 3)	-2.25%	0.466
Yes	43 (37 to 49)	45 (36 to 50)	0 (-4 to 5)	0.00%	
TC					
Sex					
Male	192 (166 to 220)	192 (165 to 225)	0 (-10 to 10)	0.00%	0.442
Female	198 (171 to 230)	210 (177 to 237)	2 (-8 to 19)	1.25%	
Age					
< 45	204 (182 to 231)	210 (184 to 231)	1 (-11 to 17)	0.57%	0.502
> 45	188 (158 to 220)	192 (160 to 225)	1 (-7 to 11)	0.48%	
DM drug					
Insulin	201 (160 to 231)	205 (165 to 235)	0 (-10 to 15)	0.00%	0.482
OHA	192 (170 to 221)	198 (174 to 229)	1 (-8 to 12)	0.71%	
BMI					
Normal	191 (160 to 220)	192 (166 to 225)	1 (-6 to 10)	0.74%	0.490
Overweight	198 (174 to 230)	210 (176 to 235)	2 (-10 to 19)	1.32%	
Obese	195 (168 to 225)	199 (167 to 222)	0 (-10 to 10)	0.00%	
Lipid lowering agent					
No	190 (166 to 220)	194 (168 to 222)	1 (-7 to 10)	0.61%	0.500
Yes	201 (174 to 230)	210 (170 to 235)	1 (-10 to 19)	0.43%	

* Mann-Whitney-U test; OHA: Oral hypoglycemic agents, TG: triglyceride, LDL-C: low-density lipoprotein cholesterol, HDL-C: high-density lipoprotein cholesterol, TC: total cholesterol

## Discussion

Our study suggests little difference between fasting and random lipid profiles of T2DM patients in Bangladesh. The difference in LDL-C and HDL-C between fasting and random state was neither statistically nor clinically significant. On the other hand, although the difference in TG (absolute difference 26 mg/dl) and TC (absolute difference 1 mg/dl) showed statistical significance, there is little value in these low differences in clinical practice.

Several previous studies, including the Copenhagen General Population Study, the Women’s Health Study in the USA, the National Health and Nutrition Examination Survey in the USA, etc., explored the possibility of establishing the random lipid profile as an alternative to the fasting lipid profile [5–7, 16, 17]. These studies reported an average postprandial change of + 26.6 mg/dl for TG, − 7.7 mg/dl for LDL-C, − 3.8 mg/dl for HDL-C, and − 7.7 mg/dl for TC compared to the fasting state. These values mostly corroborate our finding of a range of differences of 9 to 64 (median 26) mg/dl for TG, -9 to 7 (median − 1) mg/dl for LDL-C, -4 to 3 (median 0) mg/dl for HDL-C and − 10 to 13 (median − 1) for TC. In addition, another study including a similar population to ours (Asian T2DM patients) reported an average change of 42.5 mg/dl in TG, -5.8 mg/dl in LDL-C, -0.4 mg/dl in HDL-C and − 1.7 mg/dl in TC levels in the postprandial state compared to fasting, which is somewhat similar to our findings [13]. In our study, LDL-C levels were directly measured rather than calculated from Friedewald’s formula. According to the results from most of the studies, including ours, one random level of LDL-C was slightly lower than the fasting level. However, it possibly has little clinical interest [5, 7, 13]. As the level of TG increases after a meal while the levels of TC and HDL-C change little [18], the LDL-C level, when calculated by Friedewald’s formula used in most laboratories, would fall. This scenario suggests that if random LDL-C is elevated, it is potentially due to a true increase in LDL-C levels and has clinical significance for therapeutic decisions.

Our study found that the difference between fasting and random levels of lipids among T2DM patients was within the total acceptable error allowed for each lipid parameter and had a good correlation between the fasting and random lipid levels (correlation coefficients were 0.793, 0.873, 0.609, and 0.780 for TG, LDL-C, HDL-C, and TC). A similar level of significant correlation between fasting and random lipid profile was also reported in previous studies conducted among T2DM patients and the general population [3, 4, 13]. Moreover, our study suggests that the difference between the fasting and random lipid profiles did not differ significantly in different age and sex groups of patients. In addition, the finding was similar irrespective of diabetic drugs (either insulin or OHAs) or lipid-lowering therapy. Although it was previously shown that lipid-lowering therapy does not affect the concordance between fasting and random lipid levels among patients with cardiovascular diseases [4], the evidence was not exclusive to T2DM patients. Moreover, there is little evidence regarding the comparison of differences between fasting and random lipid levels according to the type of hypoglycemic therapy, which was found to be similar in the present study.

T2DM patients have an elevated risk of cardiovascular diseases, further augmented by dyslipidemia. Although the fasting lipid profile has long been used as a risk predictor of cardiovascular diseases, emerging evidence suggests that random blood samples for lipid measurement do not attenuate the value of cardiovascular risk prediction [19]. The present study’s findings support the potentiality of a random lipid profile as an alternative to a fasting lipid profile.

The present study has several limitations. First, we considered only TG, LDL-C, HDL-C, and TC for fasting and random lipid profiles; other parameters, including VLDL, chylomicrons, Apo-B, etc., were not measured. In addition, lipid measurements were not done in a single laboratory, which may influence the study results. Moreover, we included T2DM patients irrespective of their glycemic status and lipid levels, which could impact the correlation between fasting and random lipid levels. Finally, we have included only patients with T2DM, which might limit the generalizability of our result for the overall population.

## Conclusions

The current study suggests a good correlation between fasting and random lipid profile with an acceptable level of agreement in T2DM patients. In addition, a small difference is present between these two lipid profiles irrespective of patients’ age, sex, BMI, and antidiabetic and lipid-lowering therapy. Hence, a random lipid profile seems a potential alternative to the fasting profile.

## Data Availability

Patient-level data will be available on request from the corresponding author.
